# Visible-light-induced addition of carboxymethanide to styrene from monochloroacetic acid

**DOI:** 10.3762/bjoc.16.38

**Published:** 2020-03-16

**Authors:** Kaj M van Vliet, Nicole S van Leeuwen, Albert M Brouwer, Bas de Bruin

**Affiliations:** 1 Van ‘t Hoff Institute for Molecular Sciences, Faculty of Science, University of Amsterdam, Science Park 904, 1098 XH Amsterdam, Netherlands

**Keywords:** ATRA, catalysis, chloroacetic acid, lactone, photoredox

## Abstract

Where monochloroacetic acid is widely used as a starting material for the synthesis of relevant groups of compounds, many of these synthetic procedures are based on nucleophilic substitution of the carbon chlorine bond. Oxidative or reductive activation of monochloroacetic acid results in radical intermediates, leading to reactivity different from the traditional reactivity of this compound. Here, we investigated the possibility of applying monochloroacetic acid as a substrate for photoredox catalysis with styrene to directly produce γ-phenyl-γ-butyrolactone. Instead of using nucleophilic substitution, we cleaved the carbon chlorine bond by single-electron reduction, creating a radical species. We observed that the reaction works best in nonpolar solvents. The reaction does not go to full conversion, but selectively forms γ-phenyl-γ-butyrolactone and 4-chloro-4-phenylbutanoic acid. Over time the catalyst precipitates from solution (perhaps in a decomposed form in case of *fac*-[Ir(ppy)_3_]), which was proven by mass spectrometry and EPR spectroscopy for one of the catalysts (*N*,*N*-5,10-di(2-naphthalene)-5,10-dihydrophenazine) used in this work. The generation of HCl resulting from lactone formation could be an additional problem for organometallic photoredox catalysts used in this reaction. In an attempt to trap one of the radical intermediates with TEMPO, we observed a compound indicating the generation of a chloromethyl radical.

## Introduction

**Figure 1:**
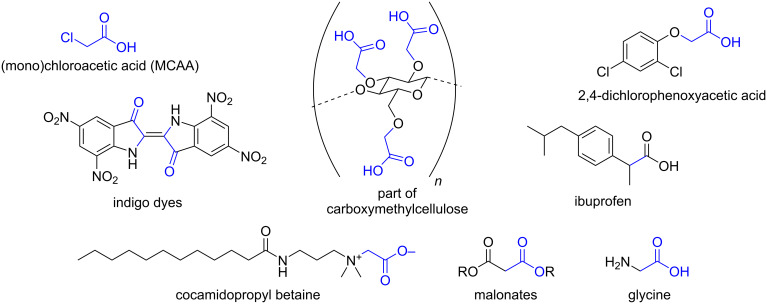
A part of the industry around monochloroacetic acid.

Monochloroacetic acid is an industrially important compound, with applications ranging from thickening agents (carboxymethyl cellulose, E466) [1–2], herbicides (2,4-dichlorophenoxyacetic acid), cosmetics (cocamidopropyl betaine), dyes (indigo vat dye) [3], to pharmaceuticals (ibuprofen, glycine, malonates) [4] (see Figure 1). Many reactions with monochloroacetic acid have been published. The production of basic building blocks like glycine [5] (>600 000 tons/year in China in 2015) and diethyl malonate from monochloroacetic acid show the value of this compound as a starting material. Many reactions with monochloroacetic acid, including the synthesis of glycine and diethyl malonate, are based on nucleophilic substitution of the chlorine [6–10].

In an attempt to find new synthetic applications of monochloroacetic acid we turned our attention to photoredox catalysis. We were inspired by the rebirth of visible light photoredox catalysis, induced by the work of Yoon, Stephenson and MacMillan [11]. By selective excitation of an organic or organometallic dye by visible light a species is formed that can act as a single-electron oxidant and single-electron reductant. In this way reactive radical intermediates can be formed catalytically in situ, resulting in reactivity different from common two-electron pathways. Photoredox catalysis reactivity is very different from traditional redox reactions, and the same reactivity cannot be achieved by stoichiometric addition of both a reductant and an oxidant to a reaction mixture (as that would lead to a rapid redox reaction between the oxidant and the reducing agent instead of converting the substrate). Excitation of the photocatalyst, on the other hand, allows continuous formation of low concentrations of both oxidized and reduced radical forms of the substrate(s), and the excited catalysts (and/or their oxidized/reduced forms) can perform both opposed redox events before returning to their original oxidation state for re-excitation. If no substrate is encountered during the lifetime of the excited state the ground state is typically regenerated.

Such photogenerated radical intermediates can be useful for the formation of new C–C bonds. Previous works from our group presented cobalt-catalyzed radical cyclization and carbonylation reactions [12–16]. Photoredox catalysis provides a way to generate radical intermediates from simple organic molecules by single-electron redox processes. Single-electron oxidation can produce carbon-centered radicals from substrates such as amines [17], alkenes [18], and carboxylates [19], or by hydrogen-atom transfer to an oxidatively formed thiolate radical [20]. Single-electron reduction produces carbon-centered radicals from, for example, aryl nitriles [21], carbonyl or imine species [22], iodonium or diazonium salts [23], or halide species [24].

The formation of radicals from halide species by photoredox catalysis has been widely studied. It has been applied as a mild method for the dehalogenation of several compounds [25–27]. In the light of photoredox catalytic C–C bond formation, its application in the field of atom transfer radical addition (ATRA) reactions is very important. Remarkably, this type of C–C bond formation became only popular in 2011, while the first example was already published by Barton in 1994 [28].

**Scheme 1:**
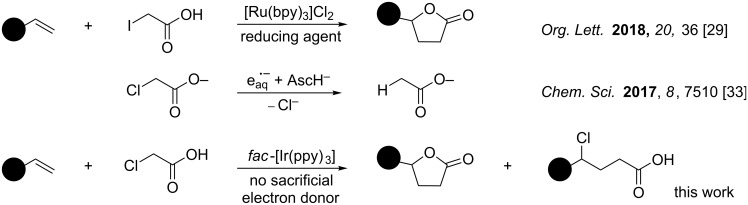
Redox based activation of haloacetic acid.

Recently, Kokotos and co-workers published a visible light photoredox catalyzed lactone formation from iodoacetic acid and alkenes [29] (Scheme 1). In addition, it has been shown that monochloroacetic acid can be reduced by hydrated electrons [30–31]. The group of Goez developed a strategy for the photoredox generation of hydrated electrons and applied that for the dehalogenation of monochloroacetic acid [32–33]. However, to the best of our knowledge, a photoredox catalyzed application of monochloroacetic acid without using a sacrificial electron donor is not known. Interestingly, monochloroacetic acid also contains a carboxyl group that could perhaps be oxidatively decarboxylated to generate a chloromethyl radical. In this study, we investigated the possibility of redox neutral, visible light photoredox catalyzed C–C bond formation with monochloroacetic acid.

## Results and Discussion

**Figure 2:**
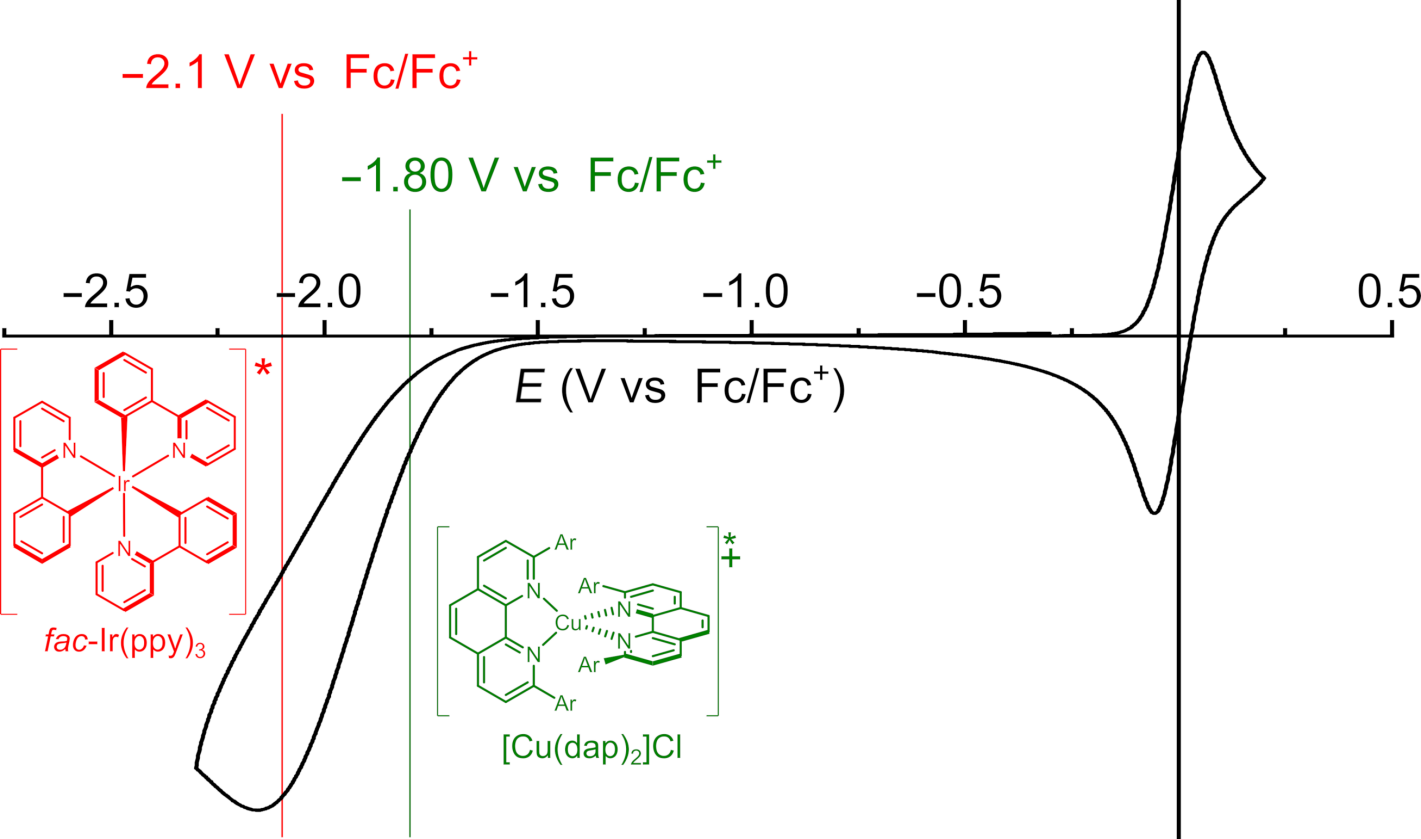
Cyclic voltammogram of monochloroacetic acid and ferrocene with 0.1 M [TBA][PF_6_] in MeCN. The potential is referenced to the Fc/Fc^+^ redox couple (0 V). The excited state reduction potentials of two different reducing photoredox catalysts (in red and green) are shown in the figure.

First, we used cyclic voltammetry to determine the oxidation potential (*E*_Ox_) of monochloroacetic acid in acetonitrile (Figure 2). This way it could be verified, if monochloroacetic acid can quench the excited state of one of the common photoredox catalysts. In Figure 1 the cyclic voltammogram of monochloroacetic acid is shown. One can see the onset potential for the reduction of monochloroacetic acid at −1.5 V vs Fc/Fc^+^. The two photoredox catalysts, *fac*-Ir(ppy)_3_ (E*_ox_ = −2.1 V vs Fc/Fc^+^) [34] and [Cu(dap)_2_]Cl (E*_ox_= −1.8 V vs Fc/Fc^+^) are strong enough reducing agents in their excited states to be oxidatively quenched by monochloroacetic acid. Based on these data, we concluded that the visible-light-promoted reductive photoredox C–C bond coupling reaction with monochloroacetic acid should be possible.

**Scheme 2:**
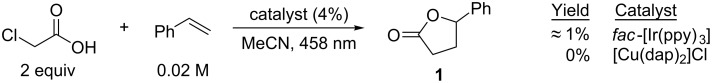
Initial attempts for lactone formation by photoredox catalysis.

As such, we chose *fac*-[Ir(ppy)_3_] and [Cu(dap)_2_]Cl for the photoredox reaction between monochloroacetic acid and styrene in acetonitrile under an atmosphere of N_2_ to form the lactone 5-phenyldihydrofuran-2(3*H*)-one (and HCl) or the acid 4-chloro-4-phenylbutanoic acid via an ATRA reaction (Scheme 2). The photoredox catalysts are excited by using blue LEDs with a peak excitation of 458 nm (see the experimental section for a detailed description) which is sufficient for the excitation of both complexes. For [Cu(dap)_2_]Cl, no product was observed, but with *fac*-[Ir(ppy)_3_] we could observe the desired lactone, albeit in small amounts.

**Scheme 3:**
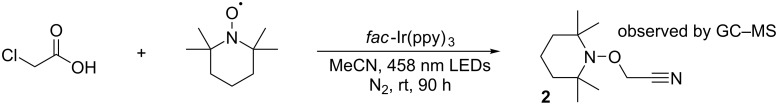
The photoredox reaction of TEMPO with monochloroacetic acid catalyzed by fac-[Ir(ppy)_3_].

After these unsatisfying results we focused on the formation of the carboxymethanide radical. We attempted trapping the radical by replacing styrene with TEMPO (2,2,6,6-tetramethylpiperidin-1-yl)oxyl). However, GC–MS analysis indicated full conversion of TEMPO to the adduct resulting from a radical coupling between a cyanomethanide radical and TEMPO (Scheme 3) for the highly reducing [Ir(ppy)_3_] (−1.73 V vs SCE in its excited state [35]). The weak C–H bond of acetonitrile (93.0 kcal mol^−1^) compared to the C–H bond strength of a carbonyl α-C–H bond (94.1 kcal mol^−1^) resulted in formation of **2** (2,2,6,6-tetramethylpiperidin-1-yl)oxyacetonitrile (compound **2**; Scheme 3) [36].

Hence, to avoid the reaction with the solvent, we applied solvents with stronger C–H bonds (see Table 1). DMF and DMSO are used frequently in the field of photoredox catalysis, but the use of these solvents did not lead to formation of the desired product either (Table 1, entries 2 and 3), using *fac*-[Ir(ppy)_3_] or [Cu(dap)_2_]Cl as the photosensitizer. Because the use of polar solvents did not lead to the desired reactivity, we turned to nonpolar solvents. Since [Cu(dap)_2_]Cl is insoluble in nonpolar solvents, we continued with the more reducing *fac*-[Ir(ppy)_3_] photocatalyst. The choice of benzene as a solvent led to a significant formation of the desired lactone, according to GC–MS analysis. The ^1^H NMR and mass spectra of the crude reaction mixture also demonstrated the formation of the ATRA product 4-chloro-4-phenylbutanoic acid. The two products were obtained in a combined yield of 49% (Table 1, entry 4). A further screening of nonpolar solvents did not lead to any significant increase of the yield or selectivity (Table 1, entries 5–9).

**Table 1:** Conditions screening for the photoredox catalyzed reaction between monochloroacetic acid and styrene.^a^

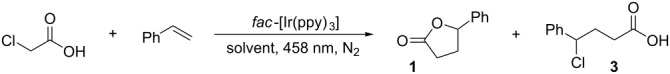				

Exp.	Cat. loading	Solvent	ClAcOH (equiv)	Yield (**1**:**3**)

1	4%	MeCN	2	≈1% (1:0)
2	4%	DMF	2	<1%
3	4%	DMSO	2	<1%
**4**	**4%**	**benzene**	**2**	**49% (1:3.0)**
5	4%	trifluoromethylbenzene	2	13% (1:4.4)
6	4%	chlorobenzene	2	22% (1:3.3)
8	4%	toluene	2	27% (1:4.7)
9	4%	DCE	2	11% (1:1.3)
**10**	**10%**	**benzene**	**2**	**84% (1:4.3)**
11	3%	benzene	2	34% (1:2.8)
12	2%	benzene	2	32% (1:2.2)
13	1%	benzene	2	18% (1:1.3)
14	0.5%	benzene	2	<1%
15	4%	benzene	0.5	46% (1:2.8)
16	4%	benzene	3	43% (1:3.3)
17	4%	benzene	4	45% (1:3.1)
18	4%	benzene	5	46% (1:2.8)
19^b^	4%	benzene	2	11% (1:1.2)
20	0%	benzene	2	–
21^c^	4%	benzene	2	–

^a^A mixture of styrene (0.02 M), monochloroacetic acid and *fac*-[Ir(ppy)_3_] in solvent (2 mL) was irradiated overnight under a N_2_ atmosphere with 458 nm LEDs. The yield was determined by ^1^H NMR spectroscopy with trimethoxybenzene as external standard. ^b^60 W fluorescent light bulb. ^c^In the dark.

The reduction of catalyst loading resulted in a lower yield and a lower selectivity for the acid compound **3** (Table 1, entries 10–14). The products indicate an oxidative quenching of the photoredox catalyst by reduction of the carbon chlorine bond of monochloroacetic acid. Varying the stoichiometry of substrates also did not lead to any improvement of the yield (Table 1, entries 15–18), most likely because the *fac*-[Ir(ppy)_3_]^+^ complex (+0.77 V vs SCE) is unable to oxidize styrene sufficiently. In the absence of the photoredox catalyst or light, the reaction does not take place (Table 1, entries 19 and 20).

**Figure 3:**
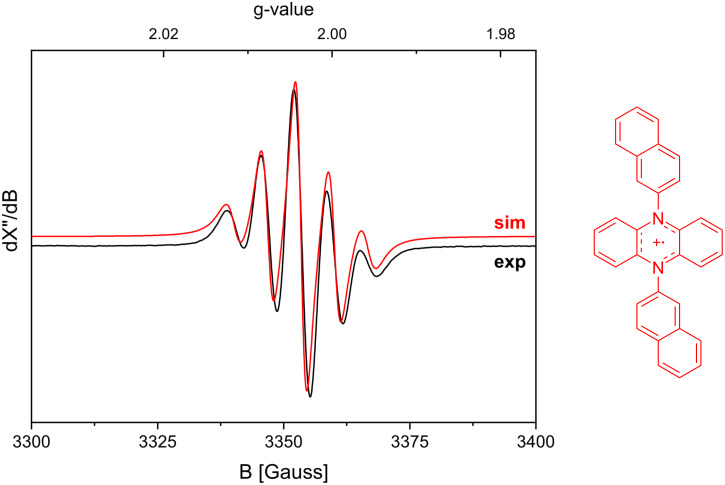
EPR spectra measured (black) and simulated (red) based on the structure of the oxidized photoredox catalyst shown on the right.

Notably, during the reaction, the bright yellow color of the solution resulting from the absorption of the catalyst disappears and an insoluble precipitate is formed. The use of C_6_D_6_ as a solvent allowed us to directly measure the ^1^H NMR spectrum of the crude reaction mixture. The presence of both monochloroacetic acid and styrene in this mixture is another indication that the catalyst had decomposed or precipitated from the solution before consumption of all substrate. Unfortunately, because of a lack of solubility, we were unable to analyze the precipitate. We also applied organic photoredox catalysts [37]. Miyake and co-workers have introduced highly reducing organic photoredox catalysts [38–43]. The use of 5,10-di(naphthalen-2-yl)-5,10-dihydrophenazine or 3,7-di(biphenyl-4-yl)-10-(naphthalen-1-yl)-10*H*-phenoxazine instead of [Ir(ppy)_3_] as the photoredox catalyst had no beneficial effect on the yield or conversion, and also led to the formation of a precipitate. However, the precipitate that was formed when the organic dye 5,10-di(naphthalene-2-yl)-5,10-dihydrophenazine was used proved to be soluble in DMSO, allowing us to characterize the material. After we discovered that the redissolved precipitate was NMR-silent, a room temperature EPR spectrum was recorded (Figure 3). The multiplicity in this spectrum is in agreement with formation of the 1e-oxidized form of the organic photocatalyst, showing hyperfine interactions with two equivalent nitrogen nuclei (*g*_iso_ = 2.0032; *A*^N^_iso_ = 18.6 MHz). The HRMS of this species showed the mass of the catalyst, thus confirming that the oxidized catalyst precipitates as a salt from solution when using benzene as a solvent.

Based on the formation of the cation of the photoredox catalyst, we believe that after oxidative quenching the catalyst is not fully reduced back to the neutral complex, accumulates in solution and as a result precipitates as chloride salt (with Cl^−^ stemming from the substrate). We assume that the same happens when using the *fac*-[Ir(ppy)_3_] photocatalyst (vide supra; although for the precipitate generated from this catalyst we were unable to redissolve the solids for characterization). Also here the catalyst is likely to precipitate as [Ir(ppy)_3_]Cl, but for this photocatalyst additional deactivation pathways could also play a role. For every lactone product that is formed, HCl is generated and in the case of *fac*-[Ir(ppy)_3_], the acid could potentially cause protodemetalation. This protodemetalation could be even more pronounced in the excited state (which is after all a metal-to-ligand charge-transfer state). Therefore, we focused on methods to trap the HCl from solution (see Table 2). An addition of 2,6-lutidine as a base leads to a lower conversion. Although the addition of a base is an efficient method to remove HCl, deprotonation of monochloroacetic acid leads to an anionic species that is harder to reduce. However, the use of 2,6-lutidine gave a 32% yield. This could be caused by the reduced Brønsted base strength in apolar solvents. Thus, to avoid using a base that deprotonates monochloroacetic acid, we added sodium chloroacetate to capture HCl from the reaction. However, under these conditions the desired product was not formed at all. Addition of [Ag]PF_6_ to remove the chloride and solubilize the oxidized photocatalyst also did not lead to any product formation. [Ag]PF_6_ is poorly soluble in benzene and the Ag^+^ ion is likely to act as an oxidizing agent quenching the excited state of the photocatalyst. Another additive we tried was tetrabutylammonium hexafluorophosphate, thought to precipitate [TBA]Cl and to keep [Ir(ppy)_3_]^+^ in solution as the PF_6_^−^ salt. However, performing the reaction in the presence of [TBA]PF_6_ led to a decreased yield. The use of diphenylurea to trap Cl^−^ did not lead to a significant increase of the reaction yield either. In addition, sodium iodide and tetrabutylammonium iodide were tested in an attempt to form an iodo intermediate which was expected to cyclize more easily to the lactone [44]. However, the lack of solubility of these reagents in benzene likely hampered nucleophilic substitution and resulted in very low yields. Sodium ascorbate was also added to investigate whether a sacrificial electron donor could increase the yields, but this was not observed. Interestingly, the reaction with sodium ascorbate favored the formation of lactone **1** over linear acid **3**. The exact reason for this behavior is not clear, but perhaps ascorbate acts as a weak base favoring lactone formation without deprotonating monochloroacetic acid.

**Table 2:** Additive screening for the photoredox reaction between styrene and monochloroacetic acid.^a^

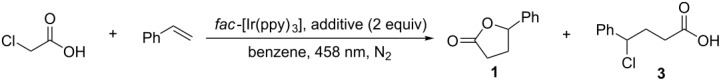		

Exp	Additive	Yield (**1**:**3**)

1^b^	2,6-lutidine	32% (1:2.2)
2	[Ag]PF_6_	<1%
3	[TBA]PF_6_	29% (1:3.8)
4	sodium chloroacetate	<1%
5	diphenylurea	50% (1:5)
6	sodium iodide	3% (1:0)
7	tetrabutylammonium iodide	≈1% (1:0)
8	sodium ascorbate	39% (1:0.75)
9^c^	MeOH	12% (1: 0.75)
10^c^	acetic acid	31% (1:2.5)

^a^A mixture of styrene (0.02 M), monochloroacetic acid (2 equiv), fac-[Ir(ppy)_3_] and an additive (2 equiv) in benzene (2 mL) was irradiated overnight under an N_2_ atmosphere with 458 nm LEDs. The yield was determined by ^1^H NMR spectroscopy with trimethoxybenzene as external standard. ^b^0.5 equiv of monochloroacetic acid were used. ^c^Additive as a cosolvent (10%).

**Scheme 4:**
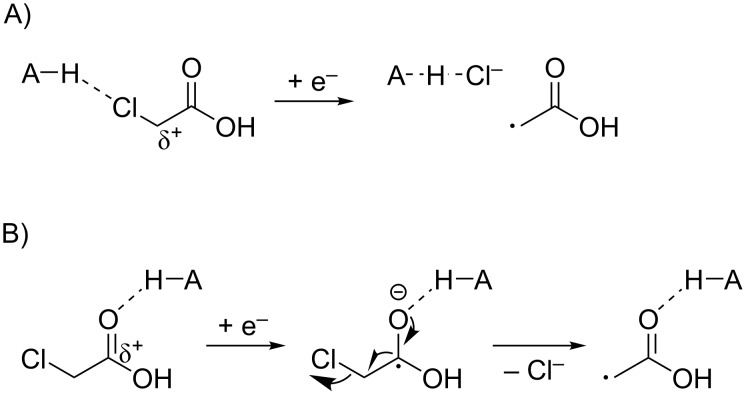
Two possible acid-assisted, reductive activation pathways of monochloroacetic acid (A–H = acid).

In another attempt to avoid the formation of HCl we applied methyl chloroacetate. Hereby the formation of the lactone is inaccessible and HCl cannot be formed. However, when using this substrate only 13% of the ATRA product were observed. Hence, there thus seems to be a positive effect of the acid on the activation of the C–Cl bond. The precise mechanism of this reductive cleavage is currently unclear. Besides the direct reduction of a carbon chlorine bond by electron transfer into the C–Cl antibonding orbital (Scheme 4A), the same intermediate can be formed by fragmentation of an α-haloketyl radical (Scheme 4B). The acidic environment could lead to easier formation of this ketyl radical. Intuitively, pathway B seems most probable.

The reaction in benzene forms mainly the acid product **3** originating from an ATRA reaction. We propose that this product is formed by radical propagation followed by a mechanism first found by Kharasch and co-workers, where the benzyl radical formed after addition of the generated α-carbonyl radical abstracts a chloride from monochloroacetic acid to obtain the product and propagates the reaction [45]. In situ IR experiments show an exponential grow for product formation, which is indeed indicative of a radical propagation mechanism (see Supporting Information File 1). In acetonitrile, a small amount of lactone was formed and no desired acid. Therefore, we investigated the effect of polarity on the selectivity of the reaction by mixing acetonitrile with benzene (Table 3). Based on the result with pure acetonitrile as a solvent (Scheme 2 and Scheme 3), we expected a lower yield with higher concentrations of acetonitrile. Indeed, the yield decreased with increasing amounts of acetonitrile. On the other hand, the selectivity for the formation of the lactone increased by adding more acetonitrile [46].

**Table 3:** Screening of the effect of mixing acetonitrile in benzene on the photoredox reaction.^a^

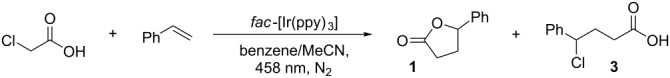		

Entry	% MeCN	Yield (**1**:**3**)

1	0	49 (1:3)
2	5	26 (1:1)
3	10	25 (1:0.79)
4	15	22 (1:0.46)
5	20	18 (1:0.28)
6	50	11 (1:0)

^a^A mixture of styrene (0.02 M), monochloroacetic acid (5 equiv), fac-[Ir(ppy)_3_] in a mixture of benzene and acetonitrile (2 mL) was irradiated overnight under an N_2_ atmosphere with 458 nm LEDs. The yield was determined by ^1^H NMR spectroscopy with trimethoxybenzene as external standard.

**Figure 4:**
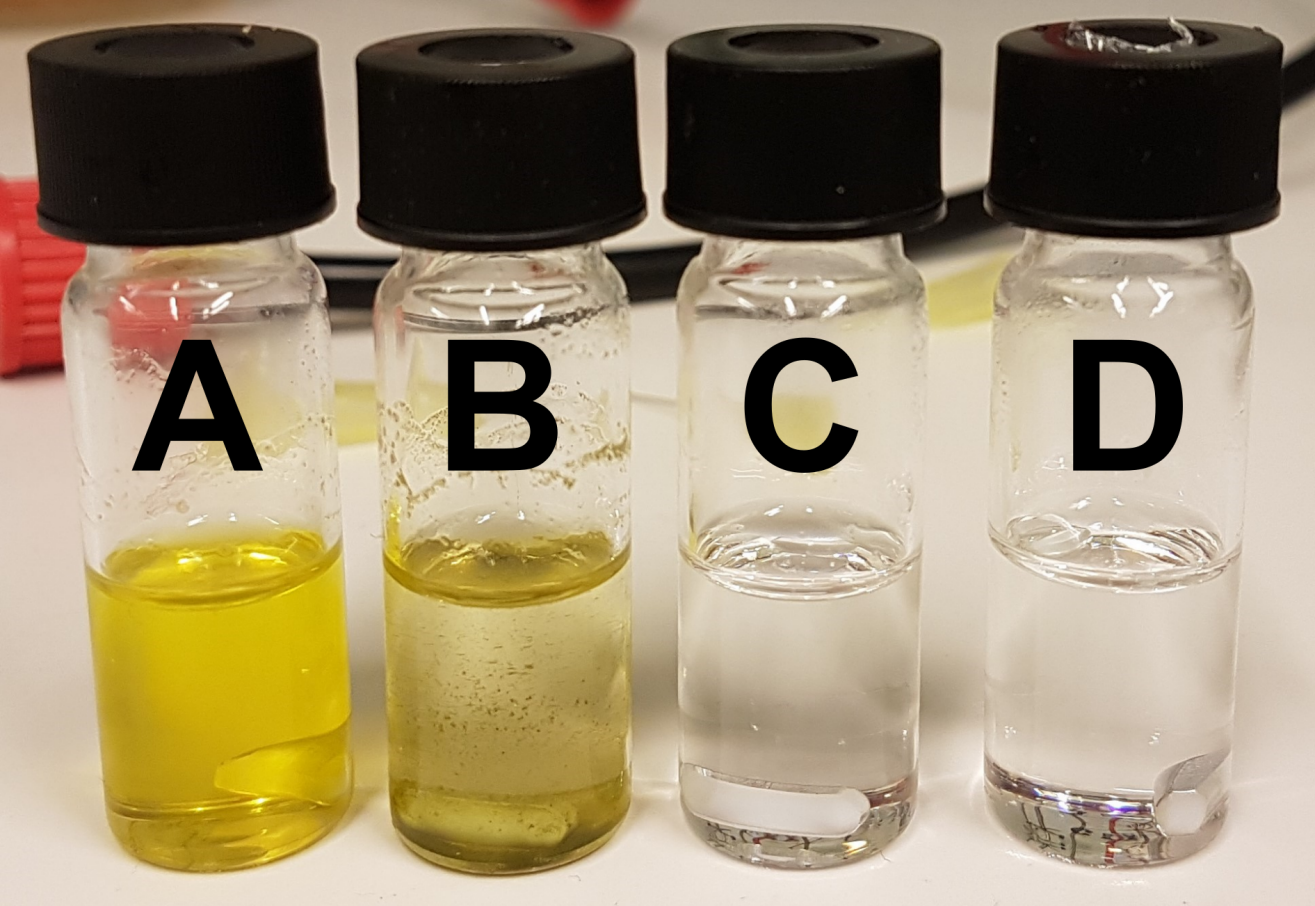
Reaction mixtures after overnight irradiation of (A) 4-chloro-4-phenylbutanoic acid (**3**) and *fac*-[Ir(ppy)_3_]; (B) **3**, *fac*-[Ir(ppy)_3_] and monochloroacetic acid; (C) **3**; (D) **3** and monochloroacetic acid. In none of the vials lactone **1** was formed.

To evaluate if γ-phenyl-γ-butyrolactone (**1**) can be formed from 4-chloro-4-phenylbutanoic acid (**3**) in benzene we reacted irradiated mixtures of **3** with either the photoredox catalyst, monochloroacetic acid or both. We did not see any formation of the lactone (**1**) after irradiating the mixtures overnight with 458 nm LEDs. Therefore, it is unlikely that in benzene direct conversion from **3** to **1** plays any significant role under the applied reaction conditions. The photoredox activation of monochloroacetic acid leads to the formation of precipitate during the reaction (see Figure 4), again indicating catalyst precipitation/deactivation.

Works in the group of Noël and others have revealed that flow chemistry can increase the rate and yield of photoredox reactions significantly [47–53]. According to the law of Lambert–Beer, only the outer shell of a batch reactor (vial or Schlenk flask) is excited efficiently. Hence, radical formation (and propagation) is only efficient within this illuminated shell. We anticipated that in a flow setup using tubing with a small internal diameter (0.71 mm) the photocatalyzed reaction pathways could be more efficient. However, in this case, performing the reaction under flow conditions did not lead to better results. The reaction performed in a flow reactor led to a combined yield of 32% within 1 hour. Furthermore, as in a normal reactor, we also observed formation of precipitate in the flow setup, forming clusters of solids. These solids presumably consist of the oxidized or otherwise deactivated photocatalyst.

**Scheme 5:**
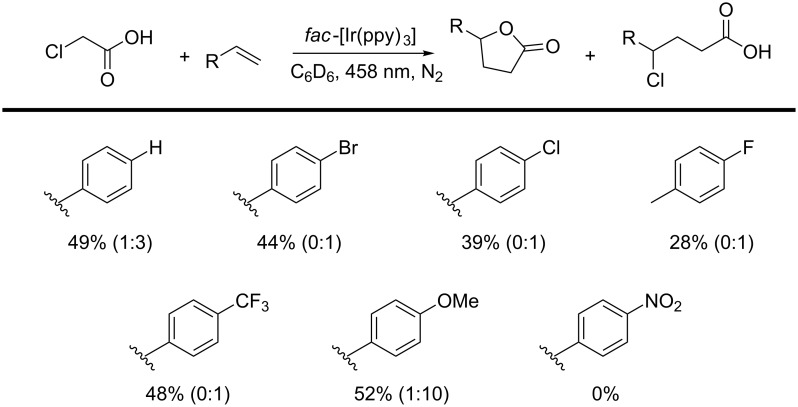
Substrate scope of styrene derivatives in the photoredox reaction with monochloroacetic acid. Yields have been determined by ^1^H NMR spectroscopy of the crude reaction mixture, using 1,3,5-trimethoxybenzene as the external standard.

More insight into the selectivity for either the formation of the lactone or the ATRA reaction was obtained by screening a small substrate scope of *para-*substituted styrene derivatives in the photocatalyzed reaction with monochloroacetic acid using *fac*-[Ir(ppy)_3_] as the photocatalyst (Scheme 5). For electron-poor *p*-halogenated and *p-*trifluoromethylated styrenes no lactone formation took place in these reactions, and only the Kharasch-addition product was observed. The benzylic radical resulting from radical addition to these styrene derivatives seems to be too electron poor for efficient oxidation induced cyclization, thus resulting in a Kharasch-type radical propagation reaction as the main pathway. Upon increasing the electron density by using 4-vinylanisole, we did see formation of the lactone product. When we used *p*-nitrostyrene, no formation of the desired products was observed at all.

**Scheme 6:**
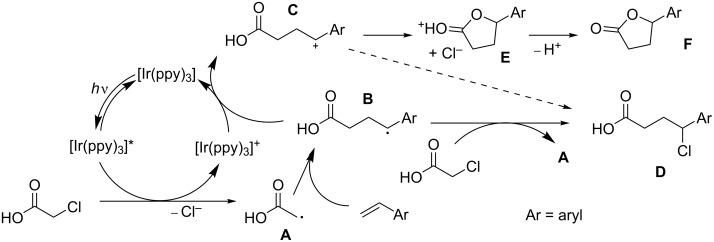
Proposed reaction mechanism.

Based on the results described above we propose the mechanism shown in Scheme 6, in which monochloroacetic acid oxidatively quenches the excited photocatalyst (*fac*-[Ir(ppy)_3_]*). The thus obtained reduced monochloroacetic acid loses Cl^−^ to give the carboxymethanide radical intermediate **A**. Addition of this species to styrene results in benzyl radical intermediate **B**. From here two pathways are considered. Species **B** can be oxidized by the [Ir(ppy)_3_]^+^ species that is formed after oxidative quenching to regenerate the ground-state photocatalyst together with the formation of the benzyl cation intermediate **C**. Species **C** then cyclizes to give intermediate **E**, which releases H^+^ to form the lactone product **F**. Alternatively, **B** forms the Kharasch-addition product **D** by chlorine atom abstraction from another monochloroacetic acid molecule, thus forming a new radical species **A**. Capture of Cl^−^ by intermediate **C** (dashed arrow) cannot be fully ruled out, but seems unlikely to compete with ring closure to **F** for entropic reasons.

**Scheme 7:**
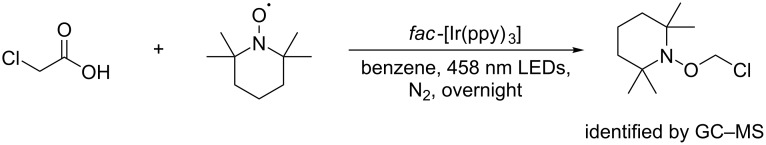
The photoredox formation of 1-(chloromethoxy)-2,2,6,6-tetramethylpiperidine.

All attempts to trap intermediate **C** using methanol or acetic acid as co-solvent (see Table 2) were unsuccessful and resulted in lower yields, especially in the case of methanol, likely due to the increased polarity of the reaction mixture. Additionally, to provide evidence for oxidative quenching, we tried trapping the carboxymethanide radical with TEMPO in benzene. We were surprised to see that instead we trapped the chloromethyl radical (Scheme 7) which was not expected in the absence of any base and with a relatively poorly oxidizing photoredox catalyst such as *fac*-[Ir(ppy)_3_]. It is known that under acidic conditions TEMPO can disproportionate into [TEMPO]^+^ and TEMPO–H [54–56]. [TEMPO]^+^ (+0.25 V vs Fc/Fc^+^ [56]) is a strong enough oxidant to oxidatively quench [Ir(ppy)_3_]* (−0.30 V vs Fc/Fc^+^ [34]). However, the oxidizing power of [Ir(ppy)_3_]^+^ (+0.77 V vs SCE) is insufficient to oxidatively decarboxylate an acetate intermediate (≈ +1.2 V vs SCE). Unexpected electron transfer pathways can be considered, such as the self-decarboxylation that was previously observed for a [Cl_3_CCO_2_H][O_2_CCl_3_] mixture [57].

Inspired by the result above on decarboxylative activation we anticipated its possibilities in cyclopropanation. The chloromethyl radical generation by photoredox catalysis is a useful strategy for cyclopropanation [58]. Most photoredox catalyzed, decarboxylative generations of carbon-centered radicals are based on the formation of “stabilized” α-amino [59–65] or benzyl [66–70] radical species. However, the generation of unstabilized alkyl radical species is also known [71–74]. Based on these observations and considerations, we attempted to use monochloroacetic acid as a precursor for cyclopropanation. We have screened a series of strongly oxidizing photoredox catalysts and various alkenes, but unfortunately we were thus far unsuccessful in obtaining cyclopropane derivatives (see experimental section).

## Conclusion

We have shown that it is possible to use monochloroacetic acid to form two types of radicals with visible light photoredox catalysis. The reductive formation of the carboxymethanide radical could be used for lactone formation with styrene. Benzene was found to be the best solvent for this reaction. However, the reaction is associated with some unsolved problems. The carboxylic acid group seems to be advantageous for the activation of monochloroacetic acid, but the apolar solvent in combination with formation of HCl is likely to cause catalyst deactivation/precipitation. This effect is more pronounced if electron-poor styrenes are used. We also found indications that a chloromethyl radical can be formed with photoredox catalysis from monochloroacetic acid, but we were not yet able to apply such reactions in a productive manner, such as in cyclopropanation reactions.

## Supporting Information

File 1 Experimental details.
