# Supplementary material for: Visible-light-induced addition of carboxymethanide to styrene from monochloroacetic acid
Source: Beilstein J Org Chem. 2020 Mar 16;16:398–408. doi: 10.3762/bjoc.16.38 (PMC7113555; doi:10.3762/bjoc.16.38)
Supplement: File 1 — Experimental details. [file Beilstein_J_Org_Chem-16-398-s001.pdf]

BEILSTEIN JOURNAL OF ORGANIC CHEMISTRY

Supporting Information

for

Visible-light-induced addition of carboxymethanide to styrene from monochloroacetic acid

Kaj M. van Vliet, Nicole S. van Leeuwen, Albert M. Brouwer and Bas de Bruin

Beilstein J. Org. Chem. **2020,** *16,* 398–408. doi:10.3762/bjoc.16.38

Experimental details

License and Terms: This is a supporting information file under the terms of the Creative Commons Attribution License (https://creativecommons.org/licenses/by/4.0). Please note that the reuse, redistribution and reproduction in particular requires that the authors and source are credited.
The license is subject to the *Beilstein Journal of Organic Chemistry* terms and conditions: (https://www.beilstein-journals.org/bjoc)

General information

All reactions were performed under an atmosphere of nitrogen or argon, unless stated otherwise. Solvents were dried by distillation over sodium (toluene), CaH_2 (acetonitrile) or by addition of molecular sieves (3 Å). Monochloroacetic acid was dried in vacuo with P_2O_5 and was stored in a nitrogen-filled glovebox. Styrene and its derivatives were filtered over activated basic alumina directly before use. NMR spectra (^{1}H and ^{13}C) were recorded on a Bruker AV400, AV300, DRX 500 or DRX 300 spectrometer; chemical shifts were referenced to residual solvent resonance signals. Mass spectrometry was performed on a JEOL JMS-T100GCv AccuTOF 2012 (FD). Emission spectra of the light sources used were measured with an ILT950 Spectroradiometer from International Light Technologies. IR spectra were recorded with a ReactIR 15 MCT infrared detector from Mettler Toledo. The detector was connected to a SiComp probe (silicon) with a 6 mm × 1.5 m silver halide fiber.

Synthetic procedures

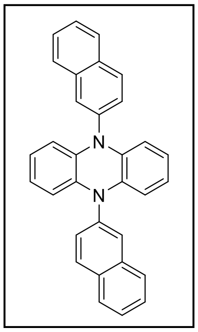

5,10-Di(2-naphthyl)-5,10-dihydrophenazine [1]

In a 50 mL flame-dried Schlenk flask, 2-bromonaphthalene (807 mg, 3.9 mmol, 1.4 equiv) was placed and the flask flushed with N_2, after which dry THF (5 mL) was added. The solution was cooled to −78 °C and *n*-butyllithium (2.5 M in hexanes, 1.6 mL, 4 mmol, 1.4 equiv) was added to give a yellow suspension. To this was added a solution of phenazine (500 mg, 2.77 mmol) in dry, degassed toluene (500 mL, 2.77 mmol). The resulting brown suspension was allowed to warm to room temperature and stirred for 1.5 h. The reaction mixture turned red after quenching with degassed water (0.2 mL) and was transferred under N_2 to a

Schlenk flask charged with $MgSO_4$. By cannula filtration, the solution was added to a flame-dried 100 mL Schlenk flask containing 2-bromonaphthalene (431 mg, 2.08 mmol, 0.75 mmol), [Pd(OAc)$_2$] (12.3 mg, 0.055 mmol, 2 mol %), [(*t*-Bu)$_3$PH]BF_4 (64.4 mg, 0.22 mmol, 8 mol %) and sodium *tert*-butoxide (410 mg, 4.3 mmol, 1.5 equiv). The reaction mixture was refluxed overnight under a N_2 atmosphere. The reaction mixture was allowed to cool to room temperature, concentrated in vacuo and purified by flash column chromatography to give the product as yellow solid (310 mg 0.71 mmol, 26% yield). ^{1}H NMR (500 MHz, Benzene-*d*$_6$) δ 7.70 – 7.65 (m, 3H), 7.60 (d, *J* = 8.1 Hz, 2H), 7.52 (d, *J* = 8.0 Hz, 2H), 7.31 (d, 2H), 7.26 (t, *J* = 7.4 Hz, 2H), 7.21 (t, *J* = 7.4 Hz, 2H), 6.30 – 6.24 (m, 4H), 5.89 – 5.82 (m, 4H).

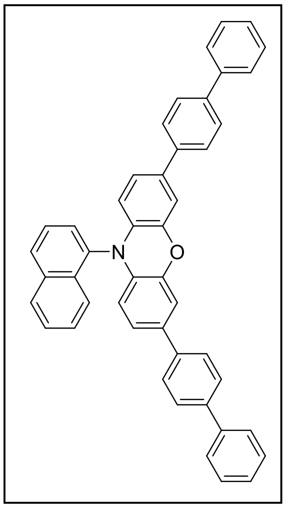

3,7-Di(4-biphenyl)-1-naphthalene-10-phenoxazine [2]

To a 100 mL flame-dried Schlenk flask was added phenoxazine (1 g, 5.46 mmol), [Pd(OAc)$_2$] (24.5 mg, 0.11 mmol, 2 mol %), [(*tert*-Bu)$_3$PH]BF_4 (158.3 mg, 0.55 mmol 10 mol %) and sodium *tert*-butoxide (1 g, 11 mmol, 2 equiv). After purging the flask with N_2, dry and degassed toluene (60 mL) and 1-iodonaphtalene (0.86 mL, 6.0 mmol, 1.1 equiv) were added. The reaction mixture was refluxed overnight (18 h) under an N_2 atmosphere, after which it was cooled to room temperature, diluted with DCM, washed with water (3 × 80 mL), dried over $MgSO_4$ and concentrated in vacuo to give a grey solid. Flash column chromatography (20% DCM in cyclohexane) afforded 10-(naphthalen-1-yl)-10*H*-phenoxazine as grey solid (780 mg). ^{1}H NMR (400 MHz, Chloroform-*d*) δ 8.10 (d, *J* = 8.4 Hz, 1H), 8.00 (dd, *J* = 8.5, 3.0 Hz, 2H),

7.67 (t, J = 7.7 Hz, 1H), 7.57 (td, J = 6.5, 1.9 Hz, 2H), 7.50 (t, J = 7.6 Hz, 1H), 6.75 (d, J = 7.6 Hz, 2H), 6.65 (s, 2H), 6.52 (d, J = 8.1 Hz, 2H), 5.72 (s, 2H).

In a flask, 10-(naphthalen-1-yl)-10*H*-phenoxazine (700 mg, 2.26 mmol) was dissolved in a mixture of $CHCl_3$ (70 mL) and acetic acid (70 mL). The flask was covered with aluminum foil and *N*-bromosuccinimide (825 mg, 4.6 mmol, 2.0 equiv) was added in portions over 20 minutes. After stirring the solution for 2 h at room temperature the solvent was evaporated. The crude was dissolved in DCM (100 mL) and washed with water (3 × 100 mL), brine (100 mL), dried over $MgSO_4$ and concentrated in vacuo to give 3,7-dibromo-10-(naphthalen-1-yl)-10*H*-phenoxazine as solid that was used further without any purification.

To a flame-dried 250 mL Schlenk flask was added 3,7-dibromo-10-(naphthalen-1-yl)-10*H*-phenoxazine (500 mg, 1.1 mmol) and [1,1'-biphenyl]-4-ylboronic acid (853 mg, 4.3 mmol, 4 equiv). Then, degassed THF (40 mL) and an aqueous solution of K_2CO_3 (2 M, 13.5 mL) were added. The reaction mixture was heated to 80 °C, a solution of $[Pd(PPh_3)_4]$ (187 mg, 0.16 mmol, 15 mol %) in THF (40 mL) was added and the reaction mixture was refluxed under a nitrogen atmosphere for 39 h. Then, the reaction mixture was cooled to room temperature and concentrated in vacuo, after which DCM (100 mL) was added. The resulting mixture was washed with water (2 × 100 mL) and brine (100 mL), dried over $MgSO_4$ and concentrated in vacuo to give a red solid. Purification by flash column chromatography (10–50% DCM in hexanes) afforded 3,7-di(4-biphenyl)-1-naphthalene-10-phenoxazine (352 mg, 0.57 mmol, 12% yield) as yellow solid. ^1H NMR (500 MHz, Benzene-d_6) δ 8.17 (d, J = 8.4 Hz, 1H), 7.71 – 7.63 (m, 2H), 7.55 (dd, J = 10.6,

5.6 Hz, 1H), 7.50 (d, *J* = 7.5 Hz, 4H), 7.48 – 7.40 (m, 9H), 7.35 (d, *J* = 2.1 Hz, 2H), 7.27 – 7.20 (m, 8H), 6.72 (dd, *J* = 8.3, 2.1 Hz, 2H), 5.87 (d, *J* = 8.2 Hz, 2H).

4-Chloro-4-phenylbutanoic acid

*4-Chloro-4-phenylbutanoic acid (**3**) is intrinsically unstable in polar solvents. The acid functionality readily substitutes the chloride to form γ-phenyl-γ-butyrolactone (**1**), which is fast process in polar solvents. Because of this, we were not able to purify acid **3** after catalysis. To the best of our knowledge, this compound has been reported once in 1890 [3]. To obtain spectroscopic data for this compound, it was synthesized according to that procedure.*

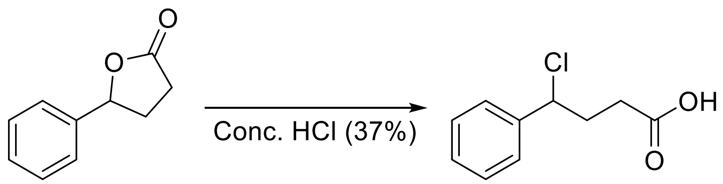

In a 20 mL glass screw cap vial γ-phenyl-γ-butyrolactone (500 mg, 3.1 mmol) was added to concentrated HCl (37%). The vial was shaken to dissolve the lactone leading to a cloudy reaction mixture, which was cooled to 0 °C to precipitate the product. The resulting white solid was obtained by filtration and dried in vacuo overnight with P_2O_5 to give the product as a white solid (481 mg, 2.4 mmol, 79%). ^{1}H NMR (500 MHz, Chloroform-*d*) δ 9.90 (s, 1H), 7.42 – 7.29 (m, 5H), 4.97 (dd, *J* = 8.6, 5.8 Hz, 1H), 2.56 (t, *J* = 7.2 Hz, 2H), 2.46 – 2.32 (m, 2H). ^{13}C NMR (126 MHz, Chloroform-d) δ 178.41, 140.88, 128.77, 128.55, 126.87, 62.32, 34.69, 31.25. HRMS (FD$^+$) m/z calc. for $[C_{10}H_{11}ClO_2]^+$ 198.0448 found 198.0432.

Light sources

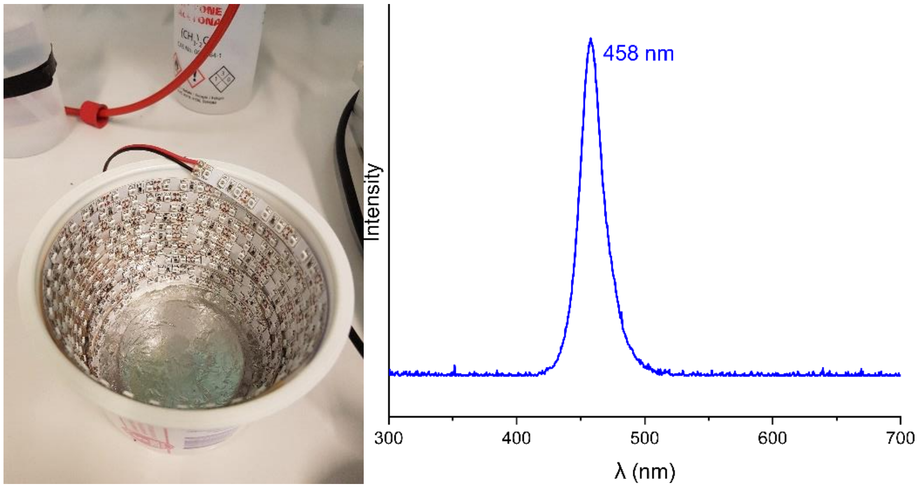

Figure S1: Top view and emission spectra of the 458 nm LED source.

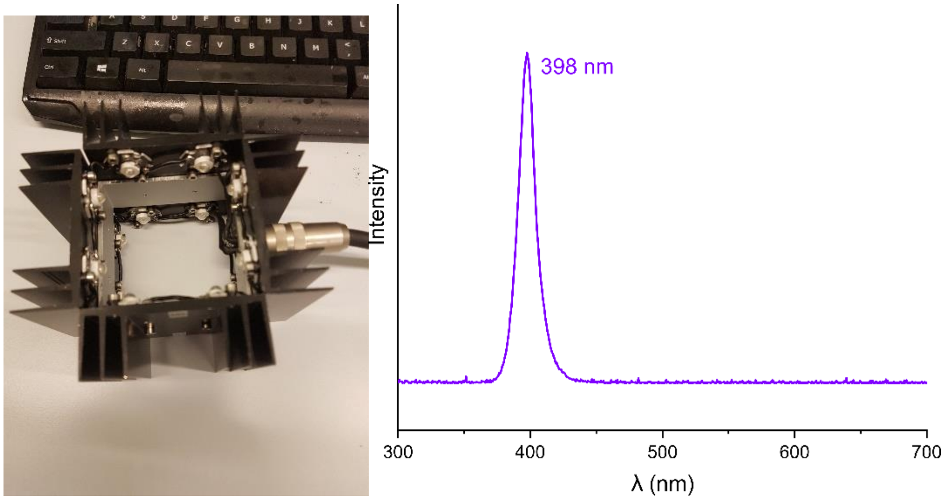

Figure S2: Top view and emission spectra of the 398 nm LED source.

Catalytic methods

Reaction in batch

A glass jar with a Schlenk flask connection was filled with eight 4 mL vials (see Figure S3). The seven outer vials are equipped with a magnetic stirring bar, the photoredox catalyst (1.6 µmol, 4 mol %), an additive (2 equiv, if applied), a septum screw cap and a small needle. The vials were evacuated for at least 30 minutes and filled with inert gas. A stock solution of monochloroacetic acid (0.04 M) and styrene derivative (0.02 M) in solvent was degassed by purging with argon for 10 minutes or by three freeze-pump-thaw cycles. Under a flow of inert gas, the stock solution (2 mL) was added to the vials containing the catalyst. The glass jar was closed under inert gas and exposed to the 458 nm light source. A fan was put on top to apply air flow for cooling. The reaction mixture was irradiated overnight. Then, the volatiles were evaporated in vacuo and 1,3,5-trimethoxybenzene was added as external standard for ^{1}H NMR analysis of the crude reaction mixture to determine the yield.

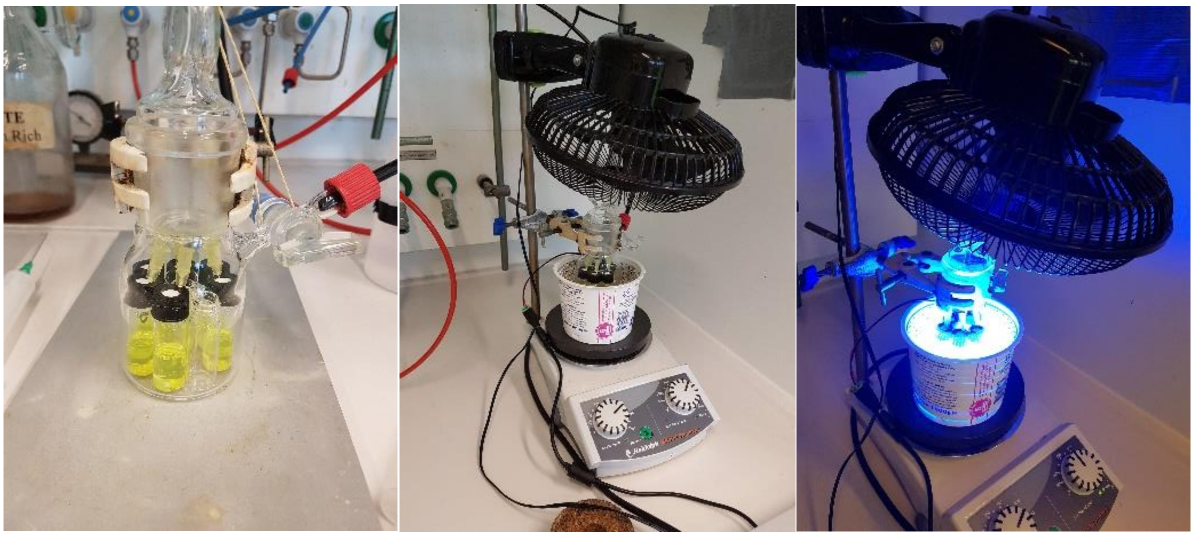

Figure S3: The glass jar with Schenk connection and the setup used for photocatalytic batch reaction.

Reaction in a Schlenk flask

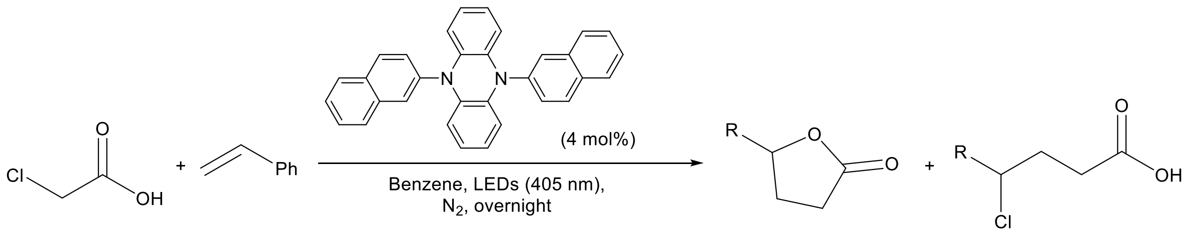

For a better absorption, we applied the 405 nm light source for the reaction catalyzed by 5,10-di(2-naphthyl)-5,10-dihydrophenazine. Because the glass jar described above does not fit in this light source, we performed the reaction in a 10 mL finger-type Schlenk flask. A flame-dried 10 mL finger-type Schlenk flask was equipped with a magnetic stirring bar and 5,10-di(2-naphthyl)-5,10-dihydrophenazine (1.4 mg, 3.2 µmol, 4 mol %). A solution of monochloroacetic acid (27 mg, 0.286 mmol) and styrene (16.5 µL, 0.144 mmol) in 7.2 mL benzene was degassed in a second Schlenk flask by three freeze-pump-thaw cycles. Of this stock solution, 4 mL were added to the catalyst. The reaction mixture was irradiated with 405 nm LEDs for 14 h, during which the yellow solution turned into an orange suspension with some black precipitate. One mL was withdrawn using a syringe, concentrated in vacuo and analyzed by ^{1}H NMR spectroscopy using 1,3,5-trimethoxybenzene as an external standard to show a 9% conversion to lactone product **1** and 8% conversion to acid product **3**. The precipitate of the resulting crude reaction mixture was obtained by filtration and dissolved in DMSO-d_6 for analysis.

Reaction in flow

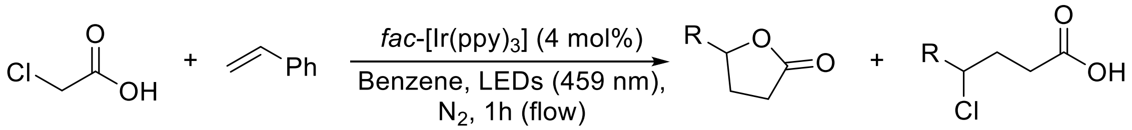

Transparent PTFE tubing (4.04 m, 0.71 mm internal diameter, 0.25 mm wall thickness) was wrapped around a 20 mL syringe with on both sides extra tubing (1.6 mL). One side was connected to a needle with a Teflon tape and the other side was inserted through a septum into a collection Schlenk flask under a nitrogen atmosphere (see Figure S4). A syringe was filled with a degassed solution of monochloroacetic acid (30 mg, 0.32 mmol, 2 equiv), styrene (18 µL, 0.16 mmol), *fac*-[Ir(ppy)$_3$] (4.1 mg, 6.4 µmol, 4 mol %) in benzene (8 mL) and attached to the tubing with the needle under a flow of N_2. The syringe with the coiled tubing was inserted into the 458 nm light source and the solution was pushed through the tubing with a syringe pump (1.6 mL/h). During the reaction we observed a decrease in color intensity and an increase in precipitate in the tubing. From the collection flask, 0.5 mL was concentrated in vacuo. Analysis by ^{1}H NMR with 1,3,5-trimethoxybenzene as external standard showed 21% conversion to acid product **3** and 11% conversion to lactone product **1**.

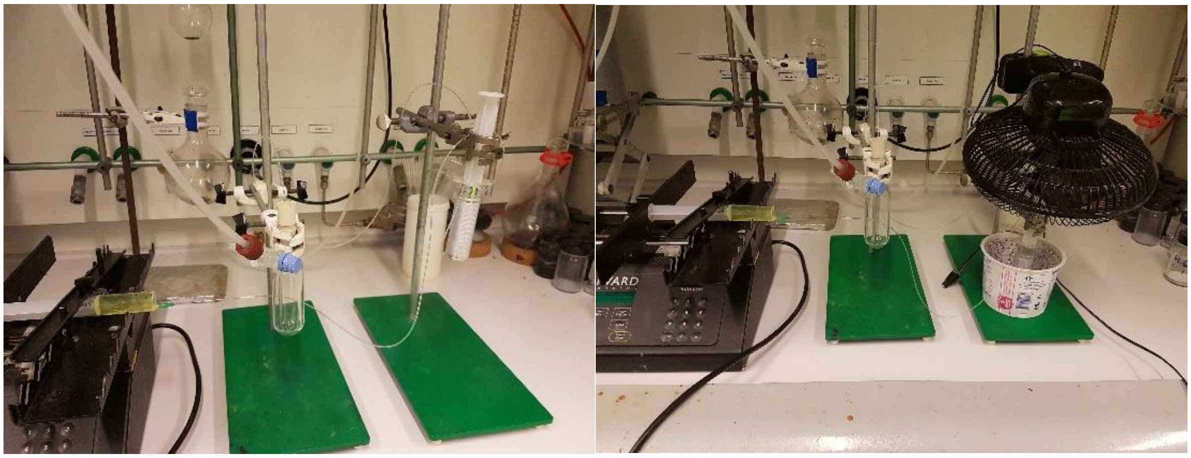

Figure S4: The setup used for photocatalysis in flow.

Cation trapping attempts

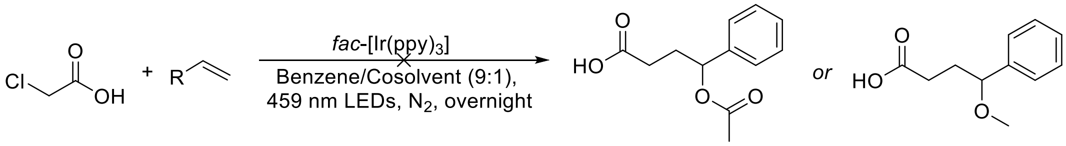

Attempts at trapping cationic intermediate **C** (Scheme 6) were performed using cosolvents, however, the trapped intermediate could not be detected by ^{1}H NMR spectroscopy. Methanol, acetic acid and a stock solution of monochloroacetic acid (0.044 M) and styrene derivative (0.022 M) in benzene were degassed by three freeze-pump-thaw cycles. The same procedure as described for reactions in a batch (see above) was used, except that 1.8 mL of monochloroacetic acid/styrene stock solution were added to the vials and 0.2 mL of cosolvent was added to reach a total reaction volume of 2 mL.

In situ IR spectrometry

Reference spectra were recorded from pure benzene and solutions of monochloroacetic acid (0.037 M), **1** (0.019 M) and **3** (0.015 M).

With ATR-IR spectroscopy we followed the reaction over time to learn more about the kinetics of the reaction (See Figure S7 for the setup used). In Figure S5 the change in IR absorption in the carbonyl region over time is shown. The shape of the spectra of the reaction mixture seems to change over time and does not resemble the sum of spectra of the isolated compounds, monochloroacetic acid, **1**, and **3**. Furthermore, negative peaks indicating monochloroacetic acid reacting away are only visible in the first 10 minutes (small dip in the kinetic profile following the peak at 1609 cm^{-1}). We suspect that interactions between **3**, monochloroacetic acid, hydrochloric acid and/or lactone **1** lead to a change in IR absorption, thus leading to overlap obscuring the negative peaks expected upon further monochloroacetic acid consumption. This is supported by the observation that we only see resonances for the substrates, and products **1** and **3** in the crude reaction mixture by ^{1}H NMR spectroscopy. The initial exponential growth of the IR peak at 1709 cm^{-1} is indicative for (partial) radical propagation playing a role in the overall mechanism, considering that the changes in IR absorption intensities resulting from non-covalent interactions are smaller than the signal increase resulting from product formation. When we followed the temperature during the exponential growth of the peak at 1709 cm^{-1} in the first 40 minutes we observed an increase in temperature from 23 °C to 29 °C. The increasing reaction rate during that time could therefore be an effect of the temperature on the rate. During the second hour of the reaction the rate appears to be constant, indicating a rate dependence on a constant factor, like the photon flux. The

decreasing rate after two hours of irradiating points to a catalyst decomposition/precipitation, a decrease in substrate concentration or a combination thereof.

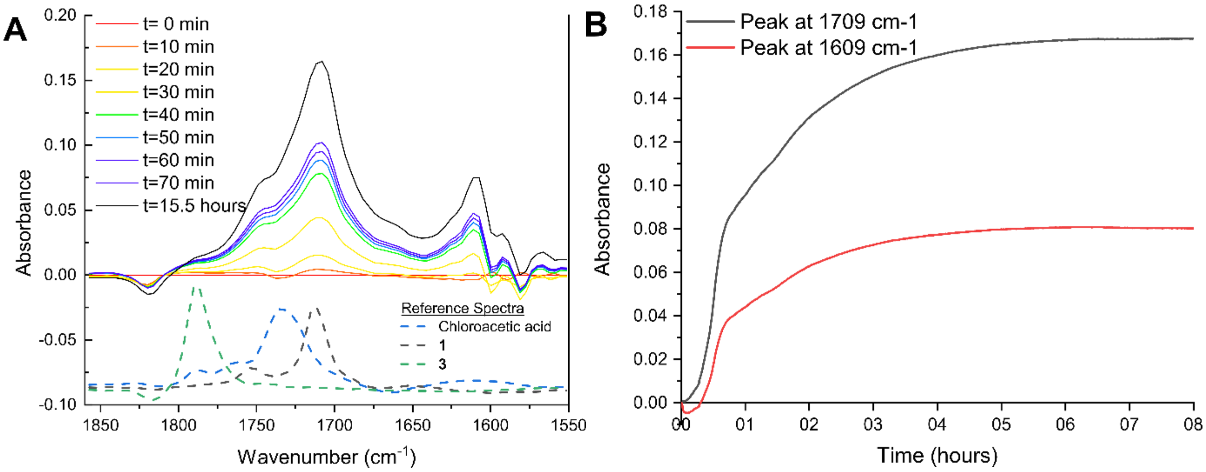

Figure S5: In situ IR spectroscopy measurement of the reaction. A: difference spectra in time relative to t = 0 and measured reference spectra of isolated compounds (offset vertically). B: Kinetic profile of the reaction based on peak height as a function of time.

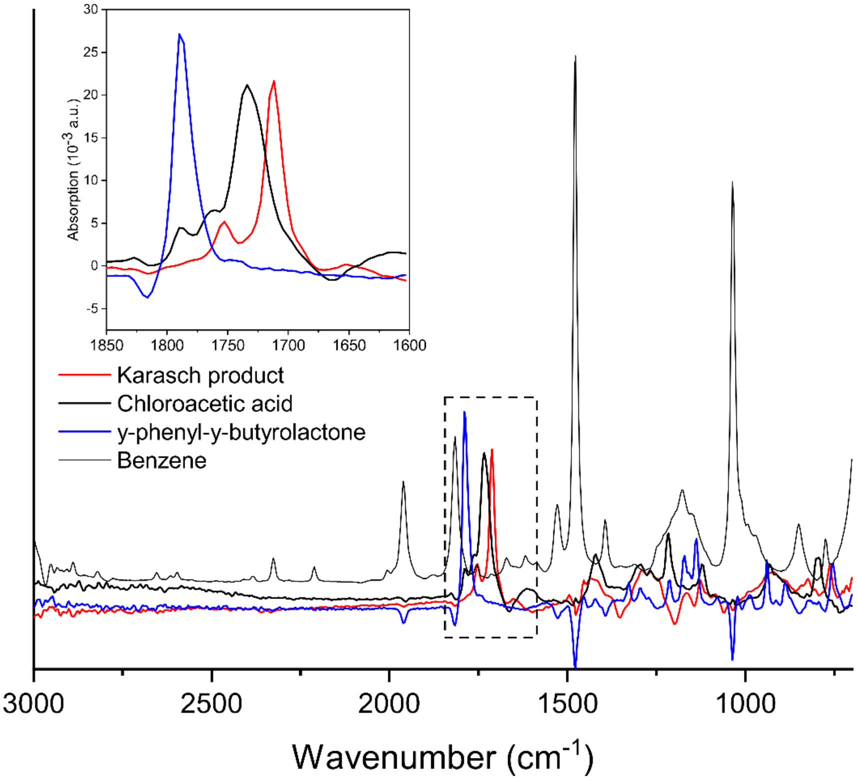

Figure S6: FTIR spectra of **1**, **3**, chloroacetic acid, and benzene.

To obtain a proper signal-to-noise ratio for the in situ IR experiment, we carried out the reaction at a 0.05 M concentration. In a Schlenk flask, a mixture of monochloroacetic acid (38 mg, 0.4 mmol, 2 equiv) and styrene (23 µL, 0.2 mmol) in benzene (4 mL) was degassed by bubbling argon for 10 minutes. The mixture was added to *fac*-[Ir(ppy)$_3$] (5.24 mg, 8 µmol, 4 mol %) in a Schlenk flask covered with aluminum foil. The yellow solution was then added to the Schlenk flask prepared with an IR probe. The sample was irradiated with the 458 nm light source and cooled with a fan (see Figure S7).

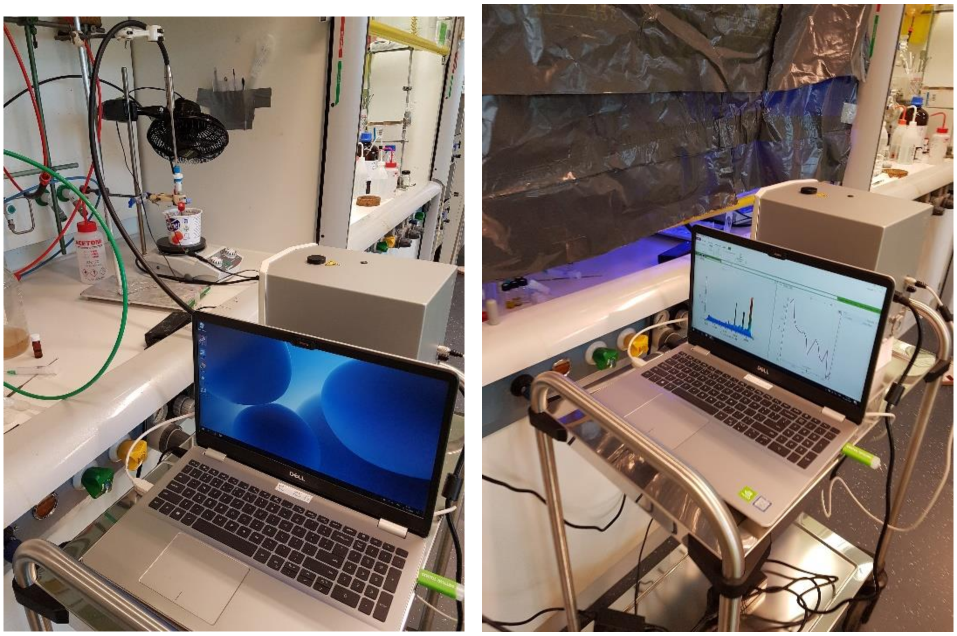

Figure S7: The setup used for in situ IR spectroscopy.

To observe the effect of hydrogen bonding in the spectra we added monochloroacetic acid (46 mg, 49 mmol) to a solution of lactone product **1** (41 mg, 0.25 mmol) in benzene (4 mL). Since both compounds have an overlapping IR band at 1788 cm^{-1} we expected accumulation of these signals. In fact, we see a decrease in absorption. Also the expected signal at 1611 cm^{-1} does not appear when adding monochloroacetic acid. This confirms that interactions between the compounds changes the shape of the IR spectra in benzene.

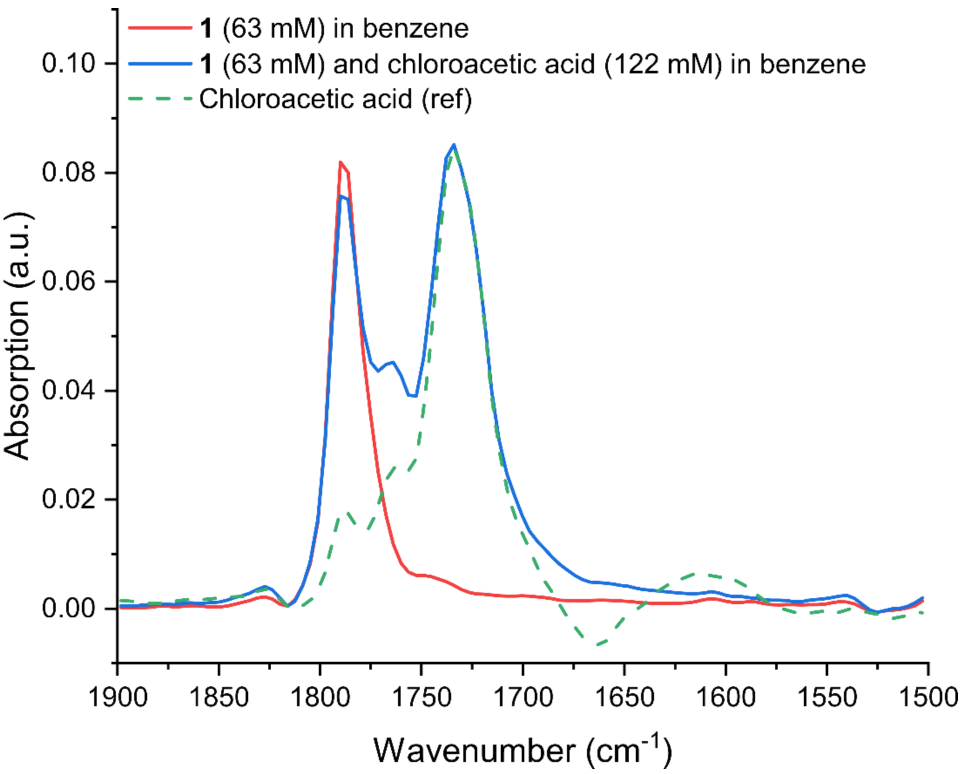

Figure S8: FTIR spectra. Red: IR spectrum of **1** in benzene. Blue: IR spectrum of the mixture from the red specrtum after addition of chloroacetic acid. Dashed green: IR reference spectrum of chloroacetic acid.

References

1. Theriot, J. C.; Lim, C. H.; Yang, H.; Ryan, M. D.; Musgrave, C. B.; Miyake, G. M. *Science* **2016**, *352* (6289), 1082–1086.

2. Pearson, R. M.; Lim, C. H.; McCarthy, B. G.; Musgrave, C. B.; Miyake, G. M. *J. Am. Chem. Soc.* **2016**, *138* (35), 11399–11407.

3. Morris, L. J. *Justus Liebigs Ann. Chem.* **1890**, *256* (1–2), 157–159.
